# Out From the Shadows – Resolution of the Taxonomy of the Family *Cryomorphaceae*

**DOI:** 10.3389/fmicb.2020.00795

**Published:** 2020-05-05

**Authors:** John P. Bowman

**Affiliations:** Tasmanian Institute of Agriculture, University of Tasmania, Hobart, TAS, Australia

**Keywords:** *cryomorphaceae*, *flavobacteriales*, *bacteroidetes*, taxonomy, genomics, Genome Taxonomy Database

## Abstract

The family *Cryomorphaceae* for many years has been a poorly defined taxonomic group within the order *Flavobacteriales*, phylum *Bacteroidetes*. Members of the *Cryomorphaceae*, apparently consisting of multiple-family level clades, have been mostly but not exclusively detected in saline ecosystems. The problems with the taxonomy of this group have stemmed from inadequate resolution of taxonomic groups using 16S rRNA gene sequences, sparse numbers of cultivated taxa, and limited phenotypic distinctiveness. The Genome Tiaxonomc Database (GTDB), which is based on normalized taxonomic ranks includes *Cryomorphaceae* as containing the genera *Owenweeksia* and *Schleiferia*. This is at odds with the official taxonomy that places these genera in the family *Schleiferiaceae*. The other *Cryomorphaceae* affiliated species have even more uncertain taxonomic positions including *Cryomorpha ignava*. To clarify the taxonomy of *Cryomorphaceae*, genomes were generated for all type strains of the family *Cryomorphaceae* lacking such data. The GTDB-toolkit (GTDB-tk) was used to place taxa in the GTDB, which revealed novelty at the family level for some of these type strains. 16S rRNA gene sequences and concatenated protein sequences were used to further evaluate the taxonomy of the order *Flavobacteriales*. From the data, the GTDB enabled successful clarification of the taxonomy of the family *Cryomorphaceae*. A number of placeholder families were given Latinized names. It is proposed that the family *Cryomorphaceae* is emended to include only the species *Cryomorpha ignava*. The family *Schleiferiaceae* is emended to account for the expansion of its membership. *Luteibaculum oceani* represents a new family designated *Luteibaculaceae* fam. nov. *Vicingus serpentipes* is the representative of *Vicingaceae* fam. nov. while *Salibacter halophilus* represents *Salibacteraceae* fam. nov.

## Introduction

The family *Cryomorphaceae*, a member of order *Flavobacteriales* within the phylum *Bacteroidetes*, was created with the description of the psychrophilic bacterial species *Cryomorpha ignava*, *Crocinitomix catalasitica* and *Brumimicrobium glaciale* (Bowman et al., 2003). Phylogenetically, the family forms a clade within the order *Flavobacteriales* that overlaps an environmental sequence clade sometimes referred to as “AGG58”, detected in seawater in one of the first molecular surveys of uncultivated marine bacteria (DeLong et al., 1993). Initially it was thought *Cryomorphaceae* were largely marine in origin, however, the description of taxa from freshwater ecosystems, such as of the genus *Fluviicola* (O’Sullivan et al., 2004, 2005; Yang et al., 2014; Dahal and Kim, 2018; Akter and Huq, 2019) instead indicates association with a wide habitat range. Bacterial species that have been cultivated and described, however, are mostly from saline ecosystems (Lau et al., 2005; Lau et al., 2006; Lee et al., 2010; Muramatsu et al., 2012; Shahina et al., 2013; Yang et al., 2013; Zhou et al., 2013; Zheng et al., 2015; Dunlap et al., 2017; Lu et al., 2017; Wiese et al., 2018). Environmental surveys have detected members of *Cryomorphaceae* in diverse marine and terrestrial systems (Pinhassi et al., 2004; Abell and Bowman, 2005; Aguilo-Ferretjans et al., 2008; Casamayor et al., 2013) but they seem largely absent from metazoan host systems.

Phenotypic data suggests cultivated *Cryomorphaceae* taxa are to some extent nutritionally fastidious requiring several amino acids and vitamins for growth. Some may exhibit photoheterotrophy (Gómez-Consarnau et al., 2019) due to the presence of proteorhodopsins. Early on, clones were found to be associated with phytoplankton in seawater samples (Pinhassi et al., 2004) as discovered in the study of DeLong et al. (1993). No specific associations with organic matter with this group are known but the taxa seem more predominant in productive ocean and coastal regions (Abell and Bowman, 2005; Fodelianakis et al., 2014; Campbell et al., 2015), amongst algal blooms (Delmont et al., 2014, 2015; Shao et al., 2020), saline waters with enhanced organic loads (Mitulla et al., 2016; Califano et al., 2017; Nilsson et al., 2018; Corsino et al., 2019), and are seemingly enriched in the marine surface layer (Zäncker et al., 2018). Along with *Flavobacteriaceae*, members of the *Cryomorphaceae* readily colonize ocean plastic waste though the biofilm communities do not exhibit preference for this type of surface with similar communities occurring on particulates and glass surfaces (Oberbeckmann et al., 2016). The wide range of habitats and diversity of this group suggests many niche and particular nutritional preferences nevertheless *Cryomorphaceae* are common enough to be detected in snow accumulating on the high Antarctic Plateau 1100 km from the Southern Ocean coast (Michaud et al., 2014).

The similarity level of the cultivated taxa of the family on the basis of 16S rRNA gene sequences ranges from 87–89% with exception of *Phaeocystidibacter* species which are more closely related to *Owenweeksia*. For many years, this level of sequence divergence was deemed reasonable for a family level clade, however, with the steady accumulation of new taxa, including description of the family *Schleiferiaceae* (Albuquerque et al., 2011), the family *Cryomorphaceae* has lost coherence (Bowman, 2015). The family *Crocinitomicaceae* was created by Munoz et al. (2016) and accommodates several former members of *Cryomorphaceae* including the recently described genus *Putridiphycobacter* (Wang et al., 2019), however, its creation only partly clarifies the taxonomy of the *Cryomorphaceae*. The Genome Taxonomic Database (GTDB, Parks et al., 2018) was used in this study to evaluate the taxonomic positions and relatedness of cultivated species of the family *Cryomorphaceae* and more broadly the order *Flavobacteriales*. The GTDB has provided a major advance to the taxonomy of bacteria and archaea by defining taxonomic ranks using a relative evolutionary divergence (RED) value. This is calculated from branch lengths generated in trees based on 120 conserved concatenated proteins, referred to as the BAC120 set. There is some compromise for taxa with unusual evolutionary rate changes, for example as exemplified by the insect endosymbionts of family *Blattabacteriaceae*, a distinct group within *Flavobacteriales*. The *Cryomorphaceae* have evolutionary rates that seem more typical for the order *Flavobacteriales* and most other bacteria, thus it was deemed the GTDB was suitable in resolving the taxonomy of the family. Furthermore, the GTDB includes the rich biodiversity comprising single cell amplified genomes and metagenome assembled genomes (MAGs). The suite of genome data incorporating *Cryomorphaceae* and its relatives in order *Flavobacteriales* is especially dominated by MAGs owing to sparse descriptions of cultivated strains.

However, it is evident the taxonomy of the GTDB should be considered to some extent provisional with the nomenclature if not with the actual taxonomy. Relevant to this study the usage of the term *Cryomorphaceae* by the GTDB and the official taxonomy – as defined by the List of Prokaryotic names with Standing in Nomenclature (Parte, 2018;^1^) is inconsistent. García-López et al. (2019) emended the family *Schleiferiaceae* to include the genera *Schleiferia* and *Owenweeksia* on the basis of whole genome comparisons. In GTDB taxonomy these genera are also grouped, however, are collectively referred to as *Cryomorphaceae* contravening rule 55 of the Bacterial Code (Lapage et al., 1990) in that legitimate names cannot be arbitrarily replaced. This family group also should include the species *Thermaurantimonas aggregans* (Iino et al., 2020). To resolve the vague state of the taxonomy of *Cryomorphaceae* and its disposition within the official and GTDB taxonomy the GTDB-toolkit (Chaumeil et al., 2019) was used to first place newly sequenced taxa within the GTDB. Some could be immediately placed in placeholder or named families. Some taxa, however, including *Cryomorpha ignava* and *Luteibaculum oceani* were indicated as having phylogenetic novelty and thus required further investigation.

## Materials and Methods

### Genome Sequencing and Annotation

Genome sequences were generated from the following strains: *Brumimicrobium glaciale* LMG 21434^T^, *Cryomorpha ignava* ACAM 647^T^, *Luteibaculum oceani* JCM 18817^T^, *Phaeocystidibacter luteus* LMG 25704^T^, *Phaeocystidibacter marisrubri* JCM 30614^T^, *Salibacter halophilus* KCTC 52047^T^ and *Vicingus serpentipes* NCIMB 15042^T^. Accession codes are shown in Supplementary Table S1 and the Data Availability section. Genomes were generated either using 150 × 2 pair end ends using the Illumina HiSeq platform or as 100 bp reads generated using the NovaSeq 6000 platform. Sequence coverage was at least 150-fold. Contigs were assembled using Unicycler 0.4.8.0 (Wick et al., 2017) and then annotated using Prokka v.1.14.5 (Seemann, 2014) as implemented in Galaxy. The GTDB-tk (Chaumeil et al., 2019) as implemented in KBase (Arkin et al., 2018) was used to place genomes in the GTDB.

### Concatenated Protein Sets

Targeted *Flavobacteriales* and reference genomes (Supplementary Table S1) were selected using the GTDB browser and by performing BLAST-P searches using chromosomal replication initiation (DnaA) protein to find more recently deposited sequences. Protein sequences were obtained from genomes downloaded from NCBI for concatenation. Two concatenated arrays of proteins were created in Geneious Prime (Biomatters Ltd., Auckland, New Zealand). The first set was based on the BAC120 set from GTDB (Parks et al., 2018). A second smaller set of proteins included a genomic region that maintains high levels of synteny across many bacterial phyla. This region typically starts with ribosomal protein S12 (RpsL) and ends with ribosomal protein L17 (RplQ). In many genomes the enolase protein follows RplQ and was thus included as was DnaA. The proteins included: DnaA, RpsL, RpsG, FusA, RpsJ, RplC, RplD, RplW, RplB, RpsS, RplV, RpsC, RplP, RpmC, RpsQ, RplN, RplX, RplE, RpsN, RpsH, RplF, RplR, RpsE, RpmD, RplO, SecY, InfA, RpsM, RpsK, RpsD, RpoA, RplQ, and Eno. This set includes 33 proteins comprising 7530 amino acid positions in total. This set was used to confirm conclusions generated from the BAC120 set and is designated here as “DnaA/RpsJ-RplQ/Eno.”

### Tree Construction and Analysis

16S rRNA sequences were downloaded from NCBI and GTDB and included those related to cultivated members of the family *Cryomorphaceae*. Near full-length sequences were used where possible, however, short sequences derived from MAGs matching those used for protein sequence comparisons were incorporated where possible. Sequence analysis was performed using NGPhylogeny.fr (Lemoine et al., 2019) and IQ-Tree (Nguyen et al., 2015; Trifinopoulos et al., 2016; Hoang et al., 2018). BIONJ-joined consensus trees were assessed with bootstrap analysis (either 200 or 1000 replicates) using default options in the given pipelines. The trees were visualized and annotated using ITOL (Letunic and Bork, 2019). Protein alignments were also analyzed using NGphylogeny-fr (ran as a Galaxy docker image) and IQ-tree using the LG model (Le and Gascuel, 2008). Consensus BIONJ trees were generated as for 16S rRNA gene sequences.

## Results

### GTDB Assignments

GTDB-tk analysis determined provisional family-level assignments for the *Cryomorphaceae* genomes sequenced (Table 1). The 3.5 Mbp MAG Gem2.bin46 derived from a soda lake metagenome (GCA_007695365; Zorz et al., 2019) was also included since it had the closest relatedness to the *Cryomorpha ignava* type strain 5.0 Mbp genome. Most of the taxa, according to the GTDB-tk were indicated to have taxonomic novelty since they were associated with families with placeholder designations including PHOS-HE28, koll-22 and 1G12. *Phaeocystidibacter* species were grouped within the family *Schleiferiaceae* while *Brumimicrobium glaciale*, as expected, was related to other *Brumimicrobium* species but is genetically distinct (average nucleotide identity score of 80.7% to *Brumimicrobium mesophilum* JCM 14063^T^). The GTDB-tk assessment of the *Cryomorpha ignava* genome placed it outside the GTDB representation of *Cryomorphaceae*. *L. oceani* and Gem2-bin46 based on GTDB-tk outputs (Table 1) also potentially form a separate family or belong to different families.

**TABLE 1:** GTDB-tk (KBase) analysis of taxa investigated in this study.

**Strain**	**GTDB-tk placement (*Flavobacteriales*)**	**RED value**	**BAC120 unique gene content (multiple copies)**	**Comment**
*Cryomorpha ignava* QSSC 1–22^T^	PHOS-HE28	0.7701	120 (0)	Taxonomic novelty determined using RED
Gem2.bin46 (soda lake metagenome)^*a*^	PHOS-HE28	0.7763	116 (1)	Taxonomic novelty determined using RED; Rnc (TIGR02191), PheS (TIGR00468), PheT (TIGR00472) not detected; PurB (TIGR00928) multiple copies
*Luteibaculum oceani* JCM 18817^T^	PHOS-HE28	0.7657	119 (0)	Taxonomic novelty determined using RED; Rnc (TIGR02191) not detected
*Vicingus serpentipes* NCIMB 15042^T^	koll-22	0.8647	120 (0)	Taxonomic novelty determined using RED
*Salibacter halophilus* KCTC 52047^T^	1G12	0.8414	120 (0)	Taxonomic novelty determined using RED
*Phaeocystidibacter luteus* LMG 25704^T^	*Cryomorphaceae*	0.7744	120 (0)	Taxonomic classification fully defined by topology
*Phaeocystidibacter marisrubri* JCM 30614^T^	*Cryomorphaceae*	0.7737	120 (0)	Taxonomic classification fully defined by topology
*Brumimicrobium glaciale* IC156^T^	*Brumimicrobium, Crocinitomicaceae*	0.986	119 (0)	Taxonomic classification fully defined by topology; SecE (TIGR00964) not detected; ANI 80.7% with *Brumimicrobium mesophilum*

*^*a*^From Zorz et al. (2019).*

### 16S rRNA Gene Sequence Evaluation

**FIGURE 1:**
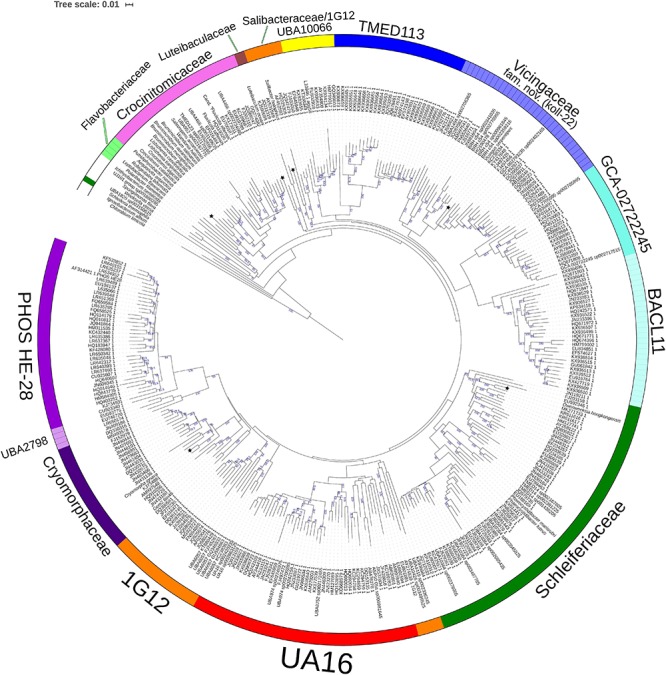
16S rRNA gene sequence based BioNJ tree focusing on sequences related to the family *Cryomorphaceae* and related taxa of the order *Flavobacteriales*, phylum *Bacteroidetes*. The tree is a consensus tree generated in IQ-Tree. Bootstrap values converted to a percentile scale are based on 1000 replicates determined using the ultrafast method of Hoang et al. (2018). Only bootstraps exceeding 50% are shown. Color strips indicate location of family-level lineages based on the GTDB framework. Family designations include the GTDB and the revised taxonomy determined in this study. Star symbols located on branches indicate validated described type strains relevant to this study. More detailed information on taxa compared is shown in Table 2 and Supplementary Table S1.

**FIGURE 2:**
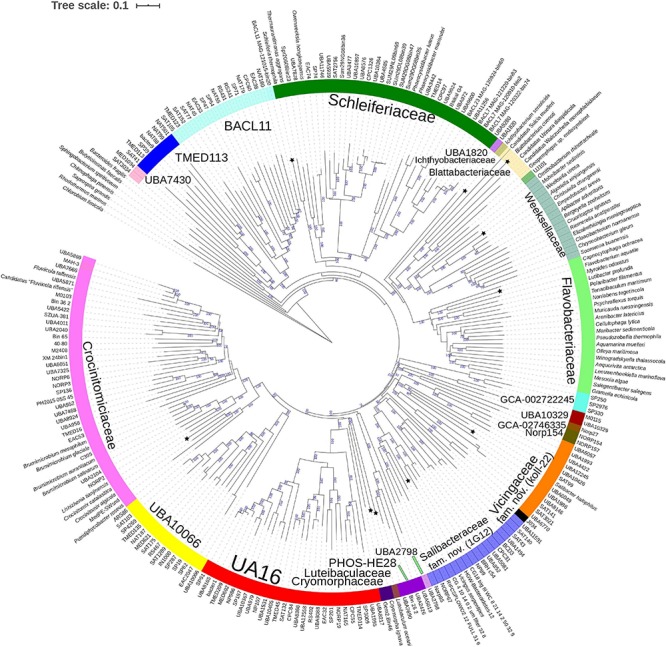
GTDB Bac120 protein set based BioNJ tree focusing on sequences related to the family *Cryomorphaceae* and related taxa of the order *Flavobacteriales*, phylum *Bacteroidetes*. The tree is a consensus tree generated in IQ-Tree. Bootstrap values converted to a percentile scale are based on 200 replicates. Only bootstraps exceeding 50% are shown. Color strips indicate location of family-level lineages based on the GTDB framework. Family designations include the GTDB and the revised taxonomy determined in this study. Star symbols located on branches indicate validated described type strains relevant to this study. More detailed information on taxa compared is shown in Table 2 and Supplementary Table S1.

**FIGURE 3:**
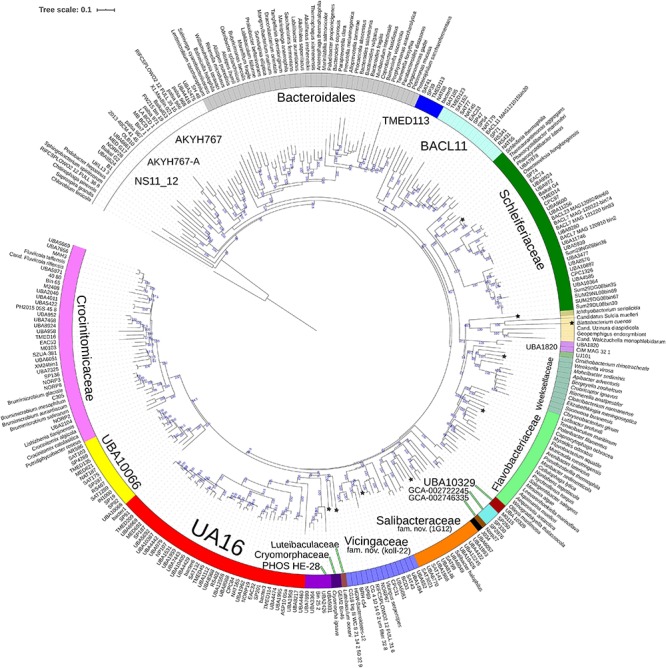
Concatenated protein based BioNJ tree covering family *Cryomorphaceae* and related taxa of the order *Flavobacteriales* and including other taxa of the phylum *Bacteroidetes*. The protein set used is based 33 proteins covering 7530 amino acid positions designated the DnaA/RpsJ-RplQ/Eno set (see “Materials and Methods” for protein list). The tree is a consensus tree generated in IQ-Tree. Bootstrap values converted to a percentile scale are based on 200 replicates. Only bootstraps exceeding 50% are shown. Color strips indicate location of family-level lineages based on the GTDB framework. Family designations include the GTDB and the revised taxonomy determined in this study. Star symbols located on branches indicate validated described type strains relevant to this study. More detailed information on taxa compared is shown in Table 2 and Supplementary Table S1.

To establish the family level relatedness of cultivated *Cryomorphaceae* taxa as well as Gem2.bin46, 16S rRNA genes and two concatenated protein sets were compared. For the 16S rRNA gene dataset most sequences related to the *Cryomorphaceae* include sequences from environmental sample surveys (Figure 1). 16S rRNA data from MAGs, as is normally the case, was relatively sparse. Family level clades are shown in the 16S rRNA tree with designations guided by GTDB, the new genome data (Figure 1) and the taxonomic revisions proposed by García-López and colleagues (2019). The family clade level designations are the same as given for all trees shown here. Within the 16S rRNA gene sequence based tree the *Cryomorpha ignava* type strain and Gem2.bin46 formed two clusters with insignificant bootstrap support. These clusters were positioned adjacent to the PHOS-HE28 and UBA2798 clades, but negligible bootstrap values occur between these clades and those designated as being *Cryomorphaceae sensu stricto*. *L. oceani* forms a distinct clade that branches deeply with no connection to other taxa. *V. serpentipes* grouped clearly within the koll-22 clade forming a lineage distinct at the genus level. *S. halophilus* 16S rRNA also formed a distinct group in terms of 16S rRNA sequences. As is shown below with protein-based trees *S. halophilus* groups with the GTDB 1G12 clade, however, this clade was polyphyletic in the 16S rRNA tree. One complication is the lack of 16S rRNA gene sequences associated with MAGS belonging to 1G12, so sequences retrieved are based largely on similarity. A low level of relatedness between members of 1G12 at the protein level (see below and Figures 2, 3) also adds potentially to the disparity observed in the 16S rRNA gene tree. Nevertheless, *S. halophilus* demonstrates a position distinct from other cultivated species. *Phaeocystidibacter* species by comparison show affiliation to *Owenweeksia hongkongensis* and its relatives.

The 16S rRNA gene sequence position of *Schleiferia thermophila* disagrees with the results from the protein alignments (Figures 2, 3). Though not shown in Figure 1, *Thermaurantimonas aggregans* also falls into the same lineage as it closely related to *S. thermophila* (sequence similarity 94%). The data from Figures 2, 3 is consistent with data from GTDB and the whole genome comparisons by García-López et al. (2019). The discrepancy in the 16S rRNA gene tree appears to reflect an aspect of the phylogeny of the *Schleiferia* clade. Since these taxa derive from thermal hot springs the rates of change in genes and proteins could differ relative to the marine-derived other members of the family *Schleiferiaceae*.

The family level clades of the *Flavobacteriales* as designated in GTDB that lack cultivated strains, otherwise formed quite distinct clusters based on 16S rRNA gene sequences including TMED113, BACL11, GCA-002722245, UA16, UBA10066, UBA2798, and PHOS HE-28 (Figure 1). This list is not complete as other minor groups not included lacked 16S rRNA gene sequence information. The UBA10066 clade includes the sequence designated “Agg58” (L10946, DeLong et al., 1993) as mentioned in the introduction.

### Protein-Level Phylogenetic Evaluation of *Cryomorphaceae*-Related Bacteria

A BAC120 protein-alignment tree was used to further investigate the relationships of *Cryomorphaceae* related taxa. A smaller protein set (designated DnaA/RpsJ-RplQ/Eno) was also tested, essentially as a means to non-orthogonally validate the relations evidenced in the BAC120 tree. This data indicated *Cryomorpha ignava*, Gem2.bin46, and *L. oceani* form a common deep cluster or form adjacent deep-branching lineages depending on the tree (Figures 2, 3). The consensus trees indicate *Cryomorpha ignava* is reliably affiliated with Gem2.bin 46, however, the connection to *L. oceani* differed between trees. An affiliation is suggested in the DnaA/RpsJ-RplQ/Eno tree (98% bootstrap) but was unsupported in the BAC120 tree. The position of these three taxa again fell adjacent to the PHOS-HE28 clade but were distinct on the basis of bootstrap values and branch distributions, which supports the 16S rRNA gene data. In any case within both protein-based trees these lineages were roughly equidistant to each other and to several other family clades including *Crocinitomicaceae*, UA16, UBA2798, GCA-002722245, and koll-22. *L. oceani* having no closely related genomes likely undergoes branch attraction into the *Cryomorphaceae* sensu stricto with the DnaA/RpsJ-RplQ/Eno protein dataset but as this dataset is expanded the relationship seems to become more ambiguous. Since BAC120 data is eight-fold greater in extent than the DnaA/RpsJ-RplQ/Eno set the BAC120 tree was considered to be more robust for final taxonomic deliberations. Phenotypic comparisons, including mol% G+C were unfortunately not helpful in these considerations due to sparse data, general inactivity in tests but also due to the fact a family level lineage would not be expected to have constrained phenotype range at least for those that are traditionally determined. Fatty acid data composition is quite different while menaquinone content is unknown for *Cryomorpha ignava*. Fatty acid profiles can also vary considerably between species within family level lineages.

For the other taxa, the affiliations are less complicated. *V. serpentipes* clustered in the midst of the koll-22 clade in both trees. Closest related MAGs include those from the BRH-c54 clade derived from rock porewater and groundwater, marine subsurface and pelagic zone metagenomes. All within this group have moderate sized genomes of around 3 Mbp and G+C contents of 32–33 mol%. This group exemplifies the habitat diversity that can occur within the family groups examined. Source information is detailed in Supplementary Table S1. *S. halophilus* is clearly a member of the 1G12 clade being centrally positioned and most closely related to MAGs SAT99, UBA6049, and UBA10426 which represent different genus-level lineages in the GTDB. The 1G12 clade includes 3 clusters that form quite deep-branches in both protein trees. The bootstrap support for the 1G12 clade was very weak (average 30%) in the DnaA/RpsJ-RplQ/Eno tree but was much stronger in the BAC120 tree (96%). Of all *Flavobacteriales* family level clades, 1G12 is perhaps the least cohesive. More genome data would be useful to confirm the memberships in this group and the overall phylogenetic structure.

*Phaeocystidibacter* species formed a distinct lineage in family *Schleiferiaceae* containing *Owenweeksia*, *Schleiferia*, *Thermaurantimonas* and two sub-clusters of MAGs from marine sources. The arrangements of these lineages were reproduced in both protein trees. The affiliation of the moderate thermophiles *S. thermophila* and *Thermaurantimonas aggregata* as the outermost members of this clade has strong bootstrap support and is congruent with the GTDB taxonomy and taxonomy based on whole genome comparisons (García-López et al., 2019).

### Overall Phylogenetic Structure of the Order *Flavobacteriales*

The protein trees also have consistent phylogenetic arrangements of 21 known family equivalent groups that GTDB assigns within *Flavobacteriales* and also reveal a possible 22nd family level member. *Crocinitomicaceae* is affiliated most closely with UBA10066 in both trees. UA16 is most closely affiliated with PHOS HE-28, UBA2798 and *Cryomorphaceae sensu stricto*, and *L. oceani* though the bootstrap support is weak in the BAC120 tree for the overall cluster. The koll-22, 1G12, GCA-002746335, GCA-002722245, and UBA10329 clades cluster together, however, bootstrap analysis does not support any meaningful specific relatedness between the clades since they deeply branch. This group also includes the hot spring MAG J034 (Ward et al., 2019; GCA-003696585.1). Its phylogenetic arrangement potentially suggests it forms another family level lineage. J034 typically branches distantly with the MAG Norp27 that belongs to GTDB placeholder family GCA-002746335. The families *Flavobacteriaceae*, *Weeksellaceae* (García-López et al., 2019) UJ101, UBA1820, *Blattabacteriaceae*, *Ichthyobacteriaceae* and *Schleiferiaceae* form a common large clade. The family represented by the xanthid crab (*Atergatis reticulatus*) gut isolate UJ101 (Yang et al., 2017) happens to be closely related to the species *Spongiimonas flava* (Yoon et al., 2013) on the basis of 16S rRNA gene sequences (Figure 1). Due to the lack of genome data for *Spongiimonas flava* and the close relatedness UJ101 has with the family *Weeksellaceae* (Figures 2, 3) creating a family for this taxon seems premature without additional information. The BACL11, TMED113 and UBA7430 clades also form a common large group – these family placeholder clades include MAGs entirely from seawater metagenomes. A summary of some features of Flavobacteriales named and placeholder families are summarized in Table 2.

**TABLE 2:** Genomic-level and habitat characteristics of family level lineages of order *Flavobacteriales* (based on data investigated in this study).

**Family designation**	**Genomes analyzed/compared**	**G+C range**	**Genome size Mbp^a^**	**Associated ecosystems**
*Cryomorphaceae* (emended)	2	40.4–49.3	3.5–5.0	Mildly saline marine and terrestrial ecosystems
*Luteibaculaceae* fam. nov.	1	39.9	2.9	Coastal seawater
*Schleiferiaceae* emended (NS9^b^)	34	36.5–57.9	>1.3–4.7 (1.8)	Seawater, marine algal cultures, riverine/lake/hot spring freshwater
*Salibacteraceae* fam. nov. (1G12, NS7^b^)	12	36.0–49.1	>2.2–5.4 (3.3)	Seawater, solar saltern, coral mucus, salt marsh/swamp water
*Vicingaceae* fam. nov. (koll-22)	17	31.0–42.8	>1.8–5.0 (3.1)	Seawater, marine biofilm, riverine/lake/aquifer freshwater
*Crocinitomicaceae* (NS6^b^)	40	31.4–49.5	>1.1–5.1 (3.6)	Seawater, saline lakes, sea-ice, marine sediment, coral mucus, seaweed surface, riverine/lake/aquifer freshwater/sediment, wastewater/bioreactor samples, soil
*Blattabacteriaceae*	5	20.9–37.0	0.2–1.3 (0.3)	Insect obligate endosymbionts
*Weeksellaceae*	14	29.1–45.1	2.0–5.6 (3.0)	Insect, animal and human microbiomes
*Flavobacteriaceae*	21	29.8–47.1	2.5–5.0 (3.8)	Marine ecosystems (seawater, sediment, fauna, flora), fish microbiomes, freshwater, soil, animal oral microbiome
BACL11	17	28.2–38.5	>1.0–3.0 (1.7)	Seawater
GCA-002722245 (NS8^b^)	3	33.9–34.7	>2.0–3.1 (3.0)	Seawater
GCA-002746335	1	41.6	>4.2	Seawater
J034	1	37.2	∼2.5	Iron-rich hot spring (Japan)
NORP154	2	40.8–43.6	>2.5–3.0	Seawater
PHOS-HE28	4	59–65	>3.2–4.5	Wastewater, activated sludge
TMED113	7	28.0–31.2	>0.9–1.5 (1.5)	Seawater
UA16 (NS10^b^)	32	37.4–61.1	>1.4–2.9 (2.3)	Seawater; lake water
UBA1820	2	47.9–56.6	>1.8–2.0	Avian and human gut microbiomes
UBA2798	1	41.8	>3.8	Activated sludge
UBA7430	2	26.9–38.8	>1.7–1.8	Seawater
UBA10066 (“Agg58” clade)	15	29.5–41.0	>0.9–2.8 (2.3)	Seawater
UBA10329	2	42.1–49.4	>1.8–2.9	Marine water and sediment
UJ101	1	30.7	3.1	Marine fauna

*^*a*^Genome size in relation to MAGs is based on genomes that have at least 90% completeness. ^*b*^Affiliation of NS (North Sea seawater) clades as designated by Alonso et al. (2007).*

## Discussion

One of the main taxonomic issues with the order *Flavobacteriales* has been the inability to confidently designate family level ranks due to a lack of understanding of its biodiversity and phylogeny. The *Cryomorphaceae* group with its lack of distinctive phenotypes and sparse descriptions hindered making meaningful taxonomic decisions. The rapid expansion of metagenome information, highly relevant for this group of bacteria was instrumental since the phylogenetic structure becomes much more resolved with the inclusion of MAGs. A number of comparisons were used in this study – 16S rRNA genes, the BAC120 and the DnaA/RpsJ-RplQ/Eno protein sets - all of which largely support the conclusions made here. The BAC120 is a highly suitable standardized protein set and when combined with data analysis as provided by GTDB-tk allows identification of novel taxa. The evaluation of a smaller, more manageable protein set should be taken as a confirmatory approach and that mirrored the phylogenetic structure achieved with the BAC120 set. Bootstrap values for some groups, such as the IG12 clade (Figures 2, 3) are less supported by the reduced information level of the DnaA/RpsJ-RplQ/Eno set. Nevertheless, using these and equivalent datasets complemented by whole genome comparisons will continue to improve the taxonomy considering the continued sequencing of type strains and discovery of new isolates and MAGs filling in remaining taxonomic “gaps.”

The analyses provide evidence for the creation of new families within the order *Flavobacteriales* and designating Latinized names to placeholder GTDB families. In particular, the data provides evidence that *Cryomorpha ignava*, the type of family *Cryomorphaceae* forms a distinct family lineage. Gem2.bin46 a MAG derived from a soda lake located within British Columbia, Canada (Zorz et al., 2019) was the closest relative with genome data. Its membership to the family was confirmed by protein comparisons though additional genome sequences would be useful for further understanding of this family given 16S rRNA gene sequences evidences a rich diversity (Figure 1). The lack of such data leads *L. oceani* to have a more ambiguous taxonomic situation. Based on the BAC120 tree as well as the more dubious support of 16S rRNA gene sequence data the decision is to place this species into its own family level group. All the other taxa analyzed could be readily inserted into placeholder or named families within the GTDB taxonomy.

Family *Cryomorphaceae* must be emended to only include the cultivated species *Cryomorpha ignava*, based on rules 21a, 23a, and 23b of the Bacterial Code (Lapage et al., 1990). Similarly, the deep position of *L. oceani* leads to the proposal it also forms a clade distinct at the family level and thus it is proposed as the type representative of the family *Luteibaculaceae* fam. nov. The position of *V. serpentipes* within the koll-22 placeholder family results in this clade being named *Vicingaceae* fam. nov. Similarly, *S. halophilus* represents the family *Salibacteraceae* fam. nov., which effectively replaces the placeholder term 1G12. Additional isolates and metagenomes with 16S rRNA gene sequences would be ideal to further define the cohesiveness of *Salibacteraceae*. Since *Cryomorphaceae* is restricted to *Cryomorpha* the representation of *Cryomorphaceae* in the GTDB must also be altered though at this stage does not affect the official nomenclature as such. It is proposed the family *Schleiferiaceae* be again emended (García-López et al., 2019) here to incorporate common traits of the cultivated members of family and mol% G+C data of its broader membership. To be consistent the family *Crocinitomicaceae* (Munoz et al., 2016) is similarly emended. Comparative evaluation of genome contents and metabolic prediction for the other placeholder families in relation to families with cultivated taxa is required to realize their taxonomy (Chuvochina et al., 2019) in a more concrete fashion but was not implemented as part of this study.

## Taxonomic Consequences

### Emended Description of the Family *Crocinitomicaceae* (Munoz et al., 2016)

As for the current description (Munoz et al., 2016) as well as: gliding motility may occur. Metabolism is chemoorganotrophic and is either strictly aerobic or facultatively anaerobic. May form carotenoids and possess proteorhodopsin. Occur in either freshwater or marine environments and thus salt requirement may occur. Species may have complex growth requirements requiring amino acids, vitamins and other compounds for growth. Major fatty acid is iso-C_15:0_. The main respiratory lipoquinone is MK6 and/or MK7. The G+C content calculated from available genome species is around 31.4–49.5 mol%.

Includes the genera *Crocinitomix* (type genus), *Brumimicrobium*, *Fluviicola*, *Lishizhenia*, *Putridiphycobacter*, *Salinirepens*, and *Wandonia*. Also includes the following placeholder genera based on the GTDB: 40-80, SZUA-381, UBA2040, UBA4466, UBA5422, UBA6165, and UBA952.

### Emended Description of the Family *Cryomorphaceae* Bowman et al. (2003)

The genus *Cryomorpha* remains as the type genus (Bowman et al., 2003). The description of *Cryomorphaceae* is emended as follows. Gram-negative, non-spore forming, non-flagellated. Gliding motility may occur. Metabolism is strictly aerobic and chemoorganotrophic. Usually strictly halophilic. May form carotenoids and possess proteorhodopsin. Species may have complex growth requirements requiring sea-water salts, amino acids, vitamins and other compounds for growth. Fatty acids are mainly C_14_–C_16_ saturated, monounsaturated and 2-hydroxylated branched-chain fatty acids. The G+C content calculated from available genome species is around 39.9–49.3 mol%.

Includes the soda lake derived-MAG Gem2.Bin46 as a genus level lineage.

The genera *Owenweeksia*, *Phaeocystidibacter*, *Luteibaculum*, *Salibacter*, and *Vicingus* are excluded from the family *Cryomorphaceae* on the basis of phylogenetic data.

### Emended Description of the Family Schleiferiaceae Albuquerque et al. (2011) emend. García-López et al. (2019)

As described previously (Albuquerque et al., 2011; García-López et al., 2019) and including Gram-negative, non-spore forming, non-flagellated cells. Either non-motile or motile by gliding motility. Taxa can be mesophilic or moderately thermophilic. Metabolism is mostly strictly aerobic and chemoorganotrophic. Oxidase positive. Catalase activity varies. Includes marine and freshwater species thus taxa may or may not require salt for growth. Usually form carotenoids and may possess proteorhodopsin. Species usually have complex growth requirements requiring amino acids, vitamins and other compounds for growth. The major menaquinone present is MK-6. Possess phosphatidylethanolamine. Major fatty acid present is iso-C_15:0_. The G+C content calculated from available genomes is around 36.5-57.9 mol%. Member genera include *Schleiferia*, *Owenweeksia*, *Phaeocystidibacter* and *Thermaurantimonas*. The family also includes the following placeholder genera based on the GTDB: TMED14, UBA10364, UBA7878 and UBA3442.

### Description of *Luteibaculaceae* fam. nov.

Lu.te. i.ba. cu.la.ce’ae (L. neut. n. *Luteibaculum* type genus of the family; -*aceae* ending to denote a family; N.L. fem. pl. n. *Luteibaculaceae*, the *Luteibaculum* family).

Gram-negative, non-spore forming, non-flagellated. Gliding motility may occur. Metabolism is strictly aerobic and chemoorganotrophic. May require salt for growth. May form carotenoids. Species may have complex growth requirements requiring sea-water salts, amino acids, vitamins and other compounds for growth. Fatty acids include iso-C_15:__0_. The G+C content calculated from available genome species is around 40 mol%. The type genus is *Luteibaculum*.

### Description of *Vicingaceae* fam. nov.

Vi.cing.a ce’ae (L. masc. n. *Vicingus* type genus of the family; -*aceae* ending to denote a family; N.L. fem. pl. n. *Vicingaceae*, the *Vicingus* family).

Gram-negative, non-spore forming, non-flagellated. Gliding motility may occur. Metabolism is usually strictly aerobic and chemoorganotrophic. Requirement for salt varies. May form carotenoids and possess proteorhodopsin. Member species may have complex growth requirements requiring sea-water salts, amino acids, vitamins and other compounds for growth. The major menaquinones present include MK-7. The G+C content calculated from available genome species is around 31.0–42.8 mol%. The type genus is *Vicingus.*

The family includes the following placeholder genera based on the GTDB: BRH-c54, GCA-002793235, UBA11591, UBA1494, UBA1494-A, UBA5081, and UBA852.

### Description of *Salibacteraceae* fam. nov.

Sa. li.bac.te.ra.ce’ae (L. masc. n. *Salibacter* type genus of the family; -*aceae* ending to denote a family; N.L. fem. pl. n. *Salibacter*, the *Salibacteraceae* family).

Gram-negative, non-spore forming, non-flagellated. Metabolism is chemoorganotrophic with growth occurring potentially under both aerobic and anaerobic conditions. Requirement for salt for growth. May form carotenoids and possess proteorhodopsin. Species may have complex growth requirements requiring sea-water salts, amino acids, vitamins and other compounds for growth. The major menaquinones present may include MK-7. The G+C content calculated from available genome species is around 36.0–44.6 mol%. The type genus is *Salibacter.*

The family includes the following placeholder genera based on the GTDB: GCA-2705995, SHAN690, UBA10426, UBA2108, UBA4419, UBA6049, UBA6057, and UBA6770.

## Data Availability Statement

The assemblies and sequences of all genomes obtained in this study have been deposited in the National Center of Biotechnology Information under WGS (BioSample) codes: SETE01 (SAMN10779751), VOOS01 (SAMN12429134), VORB01 (SAMN12423746), WACR01 (SAMN12784467), WBVO01 (SAMN12877559), WBVQ01 (SAMN12877560), and JAAGVY0 (SAMN14069737).

## Author Contributions

JB did the research and wrote the manuscript.

## Conflict of Interest

The author declares that the research was conducted in the absence of any commercial or financial relationships that could be construed as a potential conflict of interest.

## Footnotes

1 https://lpsn.dsmz.de/

## Supplementary Material

TABLE S1 Genome data analyzed including the GTDB taxonomy, environmental sources and specific genome details.
